# Approaches for Implementing App-Based Digital Treatments for Drug Use Disorders Into Primary Care: A Qualitative, User-Centered Design Study of Patient Perspectives

**DOI:** 10.2196/25866

**Published:** 2021-07-06

**Authors:** Joseph E Glass, Theresa E Matson, Catherine Lim, Andrea L Hartzler, Kilian Kimbel, Amy K Lee, Tara Beatty, Rebecca Parrish, Ryan M Caldeiro, Angela Garza McWethy, Geoffrey M Curran, Katharine A Bradley

**Affiliations:** 1 Kaiser Permanente Washington Health Research Institute Seattle, WA United States; 2 Department of Biomedical Informatics and Medical Education, School of Medicine University of Washington Seattle, WA United States; 3 Kaiser Permanente Washington Mental Health & Wellness Services Renton, WA United States; 4 University of Arkansas for Medical Sciences and Central Arkansas Veterans Healthcare System Little Rock, AR United States

**Keywords:** user-centered design, qualitative, drug use disorders, stimulants, cannabis, opioids, primary care, mHealth, mobile phone

## Abstract

**Background:**

Digital interventions, such as websites and smartphone apps, can be effective in treating drug use disorders (DUDs). However, their implementation in primary care is hindered, in part, by a lack of knowledge on how patients might like these treatments delivered to them.

**Objective:**

This study aims to increase the understanding of how patients with DUDs prefer to receive app-based treatments to inform the implementation of these treatments in primary care.

**Methods:**

The methods of user-centered design were combined with qualitative research methods to inform the design of workflows for offering app-based treatments in primary care. Adult patients (n=14) with past-year cannabis, stimulant, or opioid use disorder from 5 primary care clinics of Kaiser Permanente Washington in the Seattle area participated in this study. Semistructured interviews were recorded, transcribed, and analyzed using qualitative template analysis. The coding scheme included deductive codes based on interview topics, which primarily focused on workflow design. Inductive codes emerged from the data.

**Results:**

Participants wanted to learn about apps during visits where drug use was discussed and felt that app-related conversations should be incorporated into the existing care whenever possible, as opposed to creating new health care visits to facilitate the use of the app. Nearly all participants preferred receiving clinician support for using apps over using them without support. They desired a trusting, supportive relationship with a clinician who could guide them as they used the app. Participants wanted follow-up support via phone calls or secure messaging because these modes of communication were perceived as a convenient and low burden (eg, no copays or appointment travel).

**Conclusions:**

A user-centered implementation of treatment apps for DUDs in primary care will require health systems to design workflows that account for patients’ needs for structure, support in and outside of visits, and desire for convenience.

## Introduction

### Background

Drug use disorders (DUDs) are prevalent and deadly worldwide [1,2]. Addiction epidemics are worsening—in 2018, 184 people a day died from drug overdose in the US, and the number of fatal-and nonfatal overdoses increased in 2020 [3,4]. However, most people with DUDs do not receive treatment [5,6]. Experts recommend that treatments for DUD should be implemented in primary care to reduce this treatment gap [7-11].

Digital treatments, such as smartphone or tablet app or websites, have been touted as a means to reach more people with effective DUD treatments [12,13]. One way to classify digital treatments is to place them on a spectrum ranging from apps that are used as self-care without the help of a clinician to apps that are fully incorporated into patients’ health care and guided by a clinician [14]. Several clinician-guided treatments for alcohol or drugs are supported by the evidence of efficacy or effectiveness [15-17]. For instance, two platforms—the Therapeutic Educational System and CBT4CBT—deliver cognitive behavioral treatment through web-based modules [15,18]. Both were initially designed by researchers to augment standard care for substance use disorder and have since been marketed to health care systems in the form of apps or websites with patient-facing and clinician-facing features [19,20].

Despite the promise of digital treatments, there is a lack of knowledge about how to optimally integrate them into routine primary care. In trials of digital treatments, including those for DUD and other health conditions, patients often fail to engage with the software, leading to null results [21,22] and failed real-world clinical trials [23]. Without adequate support to enhance motivation to engage with apps, patients prematurely stop using apps or use them rarely, thereby decreasing efficacy [21,22,24-26]. This poses design and implementation problems in primary care. Evidence suggests that patients can benefit from digital treatments if they receive extensive coaching and help to use them. In contrast, the time of patients and clinical teams is limited and filled with competing demands [27-29]. Therefore, the integration of apps into health care will need to balance patients’ desires and needs with these constraints.

### Objectives

To incorporate patient voices into the design of approaches for offering apps for DUDs in primary care, this study combined user-centered design methods [30] with qualitative research methods. Drawing from the medical informatics literature, work systems models are often used to guide the design of clinical workflows [31-33]. Central to the concept of work systems’ models are actions, people, and tools that can assist patients and health care teams in embracing health care technologies. We applied these concepts to understand patient preferences on how to introduce DUD treatment apps to patients, assist them with the app setup, and offer appropriate follow-up over time. Consistent with the principles of user-centered design [30], this study served as the first within a series of implementation science studies that will iteratively design and test the effectiveness of approaches for implementing digital treatments for DUDs in real-world health care settings.

## Methods

### Study Setting

Study participants were recruited from 5 primary care clinics of Kaiser Permanente Washington, a regional integrated health care system, in Seattle area. All clinics employed licensed independent clinical social workers with some training in DUD interventions, and several clinics had primary care providers (PCPs) who prescribed buprenorphine. Consultative addiction psychiatry was available in the health system. Specialist addiction treatment programs were available through an external care network that was contracted by the health system [34].

### Eligibility Criteria

The main eligibility criteria, assessed by phone screening, were smartphone use to ensure a basic familiarity with these devices [35] along with the presence of a past-year cannabis, stimulant, or opioid use disorder based on the Mini International Neuropsychiatric Interview for the *Diagnostic and Statistical Manual of Mental Disorders*–Fifth Edition [36,37]. During our accrual period, we revised the eligibility criteria to also include patients without a past-year opioid use disorder based on *Diagnostic and Statistical Manual of Mental Disorders*–Fifth Edition if they had been prescribed buprenorphine formulations used to treat opioid use disorder [38].

### Participant Identification and Recruitment

Between July 2018 and December 2018, we queried electronic health records to identify patients aged 18 years or more with a recent primary care visit, who had screened positive for cannabis or other drugs [39-42] or had a documented DUD diagnosis. The study staff mailed invitations with a preincentive of US $2 to 101 potentially eligible patients [43,44]. We phoned 77 of these patients for eligibility screening while providing a US $10 incentive for those who completed phone screening, regardless of eligibility. Multimedia Appendix 1 presents a participant recruitment flow diagram noting the reasons for exclusion. We used purposeful sampling [45] to promote sample diversity by race, gender, age, substance type (opioid, cannabis, stimulant), and prior DUD treatment or mutual support program attendance [46].

### Sample

The study’s initial recruitment goals were based on pragmatic and empirical criteria. We sought to capture a sufficient diversity of patient perspectives to inform the initial implementation of digital treatments in a primary care practice that could then be iteratively adjusted during implementation within a health care system. We targeted a minimum of 12 participants [47] and stopped recruitment at 14 while monitoring saturation during the analysis [48]. Among the 14 participants, 6 had opioid use disorder or were taking buprenorphine, 9 had cannabis use disorder, and 4 had stimulant use disorder (participant details in Multimedia Appendix 1).

### Semistructured Interviews

The interview guide was designed to elicit preferences regarding workflow design among candidate approaches for offering and supporting the delivery of app-based DUD treatments. The interviews utilized user-centered design tools, including personas and storyboards. Personas depicted a visual *user profile* to show participants an example of a person who might be a typical user of an app [49]. We developed three personas—one each for stimulants, opioids, and cannabis—that depicted a hypothetical patient with several DUD symptoms who was interested in learning about options for help (Multimedia Appendix 1). We presented one persona per participant. We also used storyboards [50], which used illustrated panels to show core features of candidate workflow designs.

**Figure 1:**
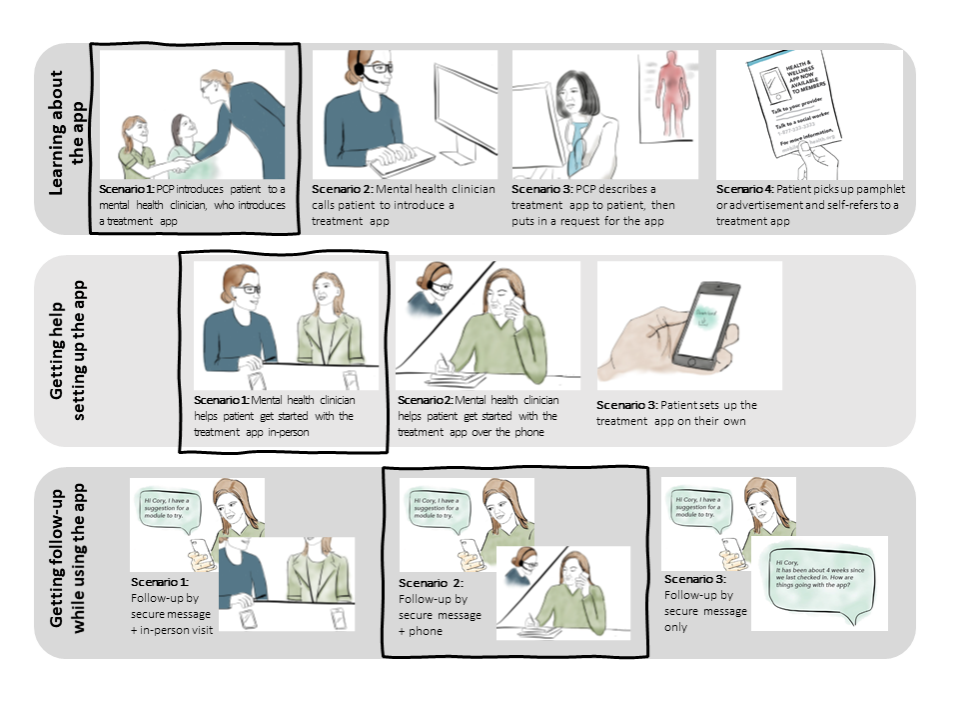
Scenarios depicting potential introduction (learning about the app), setup (getting started with the app), and follow-up (getting follow-up while using the app) phases for a drug use disorder treatment app. The boxes denote the overall workflow most preferred by participants. PCP: primary care provider.

The storyboards showed alternative scenarios to support patients’ app use. Scenarios fit into three workflow phases: (1) introducing the app to patients and helping them learn about it (*Introduction*); (2) setting up patients interested in using the app with the treatment (*Setup*); and (3) following up with patients who agree to use the app to promote engagement and execute a care plan that includes the app (*Follow-up*). There were 3 to 4 candidate scenarios as options for each workflow phase (Figure 1; complete storyboards are presented in Multimedia Appendix 1, which includes detailed information about the storyboard design process and rationale behind each scenario). Scenarios varied in whether interactions were done virtually or in person, how much help the patient received, which team members were involved, and other aspects of communication and workflow. Interviewers read aloud all scenarios within a phase, asked participants to rank scenarios according to their preferences, and probed for additional information.

Study interview materials were informed by domains of work systems models (eg, workflow actions or tasks, individuals or people, and tools that facilitate work) [33], implementation science frameworks (eg, the extent of support and how to guide patients) [14], and prior digital treatment studies (eg, privacy issues and concerns regarding the use of electronic self-reported data) [22,51-53]. The guide also drew from theory and literature on patient treatment engagement and health services design in primary care [21,22,52-63]. Clinical leaders and clinicians from Kaiser Permanente and a safety net health clinic provided input to the materials. Although not analyzed in this study, the interviews asked about background experiences (eg, app use; Multimedia Appendix 1 contains an interview guide summary). Interviews were conducted in person and digitally recorded and professionally transcribed. Interviews lasted for an average of 87 minutes, including informed consent.

The Kaiser Permanente Washington Institutional Review Board granted ethical approvals. Written informed consent was obtained from all the participants enrolled in this study. A waiver of written informed consent was obtained to conduct telephone screenings. A waiver of informed consent was obtained for the identification of potential participants using electronic health records.

### Data Analysis

For qualitative analysis, we imported transcripts into Dedoose v7.0.23 (SocioCultural Research Consultants) [64]. We applied template analyses to qualitatively code excerpts of text [65], which used a combination of inductive and deductive approaches. A priori codes were based on the interview guide topics. Codebook definitions were informed posthoc by the Workflow Elements Model [31,32] constructs to help in classifying participant preferences regarding the physical or virtual *actions* that need to be performed, the *people* (eg, clinicians) who need to perform the actions, and the physical and virtual *tools* used to promote app delivery and use. For the first 5 transcripts, 2 study team members coded them, a third study team member reconciled them, and the team discussed achieving consensus in the form of a codebook. To further increase rigor, we kept memos and an audit trail of code revisions [66,67]. The remaining transcripts were split among team members for coding, and the principal investigator (PI; JEG) reconciled them. The lead analyst (TEM) examined the fully coded data for patterns within the coding hierarchy and preliminary themes that occurred across codes, regularly meeting with the PI to review results. In several meetings, the PI and lead analyst used affinity mapping, a user-centered design activity that helps in clustering similar ideas or concepts together [68] to further refine the themes. The themes were discussed with other team members before they were finalized.

### Availability of Data and Materials

The interview materials are provided in Multimedia Appendix 1. Additional interview materials are available from the first author upon reasonable request.

## Results

### Preferences Regarding Workflow Actions

This section presents patient preferences regarding the actions performed while facilitating a DUD treatment app, by each workflow stage.

#### Introduction Stage: Learning About the Treatment App

Nearly all participants preferred to learn about app-based treatment options during an in-person primary care visit where drug use was already being discussed. Participants generally did not want to learn about an app during an unexpected *cold* call with a clinician they had not met. Cold calls in relation to drug use would feel intrusive, potentially awkward, and embarrassing. Participants who were open to receiving a phone call to learn about an app said they would at least need to know about the phone call in advance. Only 2 participants were interested in a self-guided treatment where they would learn about an app via a pamphlet without any interaction with a health care professional, and only 1 participant said that they would respond to a waiting room advertisement. A few noted that the convenience of accessing the app without help from a clinician was appealing, but others said they would not be motivated to use an app without support. As one participant explained:

I just don’t see that I would pick [the flyer] up ... It would be more effective if, after meeting with my primary care person about my addiction issues, they got me in direct contact with [a mental health clinician].

P3, who was receiving medication treatment for opioid use disorder

#### Setup Stage: Getting Started With the App

Overall, participants thought that it was critical to avoid getting “*stuck”* while setting up and starting to use an app. Having a seamless experience would be critical for maintaining motivation. One participant said the following:

Sit next to me and walk me through it. Hold my hand.

P13, participant with cannabis use disorder

Most participants preferred in-person assistance to help them get started with the app once they decided to use it. However, several others wanted to receive this help over the phone to reduce the amount of time spent at their visit or to avoid returning for a visit. For these participants, the phone setup was “the best of both worlds.” One participant said as follows:

number one is like, we agree that [persona name] will schedule an in-person appointment. In my case I’m very reluctant to do stuff like that, so okay, extra work for me–awesome, thanks. This one [solo setup scenario] is like all on my own, no support. So this one [phone setup scenario] I feel like is the best of both worlds.

P3, participant with cannabis use disorder

Still, some thought that scheduling phone appointments would be a *chore*. Few preferred to set up the app without help; these participants noted that setting up the app on their own would save time.

#### Follow-up Stage: Getting Support Over Time for Engagement and Care With an App

Half of the participants preferred follow-up to occur over the phone. This would provide personalized communication and a relationship that would help them stay accountable without the inconvenience and monetary costs of an in-person visit. Even those who favored an initial visit in person generally preferred a phone follow-up. Few preferred a follow-up strategy that only involved secure messages, but they liked the idea of receiving messages in addition to phone calls. One participant noted:

Secure messages and phone for me ... Because that way you have somebody on the phone to tell you if you’ve interpreted the message right ... I’ve had that happen many times. A doctor will say something and I don’t necessarily interpret it correctly or the way that it’s meant.

P14, participant with opioid and stimulant use disorder

Most participants thought that reading and responding to a message at a later time would be convenient, and some were more receptive to more frequent contacts if done over messages. However, others noted that they do not check messages or thought they would be “too easy to ignore.” One participant added the following:

If you’re having that reluctance, that’s so much easier to just be like well, no, I’m not going to do that.

P10, participant with cannabis use disorder

### Preferences Regarding the People Who Could Deliver Digital Treatments

Preferences regarding the people who could facilitate a DUD treatment app were often applicable across multiple stages of the workflow (Multimedia Appendix 1). Thus, the preferences are presented here for the overall workflow.

Most participants preferred a clinician with mental health expertise to introduce and guide them in app use over time. Several participants said that mental health clinicians might have more knowledge about treatment options than a PCP, more experience treating drug use, or more time to describe features of an app and walk them through the setup process. However, some preferred to only talk to PCPs and said that mental health clinicians would make it feel like their “issue was serious.” Other benefits of working with a PCP ranged from wanting to work with someone with whom they had an established rapport, wanting fewer clinicians involved in their care, and wanting to work with someone who can offer medical advice. Many acknowledged logistical constraints of working with a PCP (eg, limited time and challenge of scheduling appointments). One participant said the following:

I don’t think the primary care [provider] needs to be concerned with that. They need to have some knowledge of it, but I think the major, the main focus of it would be with the social worker, plus the social worker would be able to follow up with the patient as far as once they get started using the app. It would be easier follow up for the social worker than the primary care [provider].

P5, participant with stimulant use disorder

A few participants said it would be helpful to hear from someone with lived experience who had “been through it before” to help them decide whether to use the app over other treatment options. They did not feel that it was important to have such an individual remain involved after they had started using the app.

Finally, participants described the value of having access to technical support for help with downloading, setup, and use of the app. Participants pointed out that they would not want technical issues to consume valuable visit time. Participants clearly differentiated technical or setup assistance from treatment-related assistance and drew boundaries around the type of help they would want from each person.

### Preferences Regarding the Tools That Could Help Facilitate the Use of the Digital Treatment

Participants described a range of ideas about virtual or physical tools that could facilitate the delivery of an app (Textbox 1). For instance, pamphlets, user ratings and reviews, and trial versions of the app were suggested as tools for the introduction stage. Written instructions and video tutorials could help with the setup stage. Several tools were suggested for the follow-up stage to facilitate communication (eg, a “*Get Support”* button to help contact the care team).

Having a way to check in with your doctor about it would be useful ... In [the phone setup scenario] if there could be a way to demonstrate it like in [the in-person scenario], with a screen sharing thing – I think that would be super useful, or maybe like a video, like an instructional video showing how to navigate it, that would be useful, since you wouldn’t be able to do it in person.

P12, participant with cannabis and stimulant use disorder

**Tools that could help facilitate the use of a drug use disorder treatment app in primary care, as suggested by patients. Tools are presented by the workflow stage.**

**Introduction Stage: Learning About the Treatment App**

- Pamphlet explaining how the treatment app will benefit the patient
- User ratings and reviews that could lend credibility to the treatment app
- Trial version or demonstration of the treatment app allowing patients to test it out before committing
- Advertisements or information about treatment app on a health plan or agency website

**Setup Stage: Getting Started With the App**

- Written instructions for getting started with the treatment app or a user guide
- Video tutorial for getting started with the treatment app
- Smartphone requirements for downloading and running the treatment app (memory storage, operating system version, and needing to know their app store password)
- A webpage or button in the app that provides answers to frequently asked questions

**Follow-up Stage: Getting Support Over Time for Engagement and to Execute a Care Plan While Using the App**

- Telephone caller identification (*caller ID*) so that the patient knows if their clinician is calling (patients may not answer their phone if the incoming call looks like a generic or toll-free number)
- Contact or *Get Support* button
- Technical assistance (for app and device-related questions)
- Clinician contact information or direct messaging feature (for treatment questions)
- Screen sharing functionality so the patient’s smartphone screen can be viewed by a support professional
- Reminders and notifications, sent in accordance with the level of engagement with the treatment app (ie, more frequent reminders or notifications if patient is not using the app)

### Cross-Cutting Themes

Four cross-cutting themes emerged across codes in the codebook. Themes are described below; Textbox 2 provides representative quotes for each theme (Multimedia Appendix 1).

**Themes derived from analyses across the codebook: cross-cutting recommendations for designing a patient-centered approach for offering treatment apps for drug use disorders in primary care**

**Established Relationships and Trust Would Facilitate a Better Patient Experience**

- “It’s a person who’s already talking to her about her drug addiction, like supplying Suboxone or whatever, that is saying hey, this might help. And I feel like people would be a little bit more receptive if it’s someone that they already trust with their treatment.” (P1, participant with cannabis and opioid use disorder. Had prior drug use disorder [DUD] treatment.)
- “I mean the main thing is support...I would say the best people that will help you is if they understand what you’re going through, especially during that period, and they need to understand what you went through. It doesn’t necessarily have to be that way, but that’s how I was, and primary care provider helped me in the professional way, and then the emotional issues of that time...But I will say if someone is truly trying to help this person making them know that there’s still someone that cares about them, whatever, in any way it’s a good thing. It is. Some people have no one, and even small interactions can make a difference in positive ways.” (P2, participant taking buprenorphine whose opioid use disorder is in remission. Had prior DUD treatment.)
- “I know for me personally that if I’m talking with somebody–I mean, if you can send me something, I can more likely put it on the back burner. Whereas if I have an actual conversation with them – I don’t know, it’s just more personal. I think it’s more of a personal check in, that there’s a real human being right there that’s interested in my care.” (P3, participant taking buprenorphine whose opioid use disorder is in remission. Had prior DUD treatment.)

**Patients Were Open to Team-Based Approach When Getting Support From Their Primary Care Team in the Use of Apps**

- “I mean if there were actually social workers doing this, then I suppose that would be who I would be contacting, and I wouldn’t really have any issue with that because that’s just sort of how it would be. It would be another person I’m meeting to satisfy a different medical need that I have. I don’t have a lot of hang-ups about meeting different providers for different issues. I’ve done a lot of that in the past.” (P8, participant with cannabis and stimulant use disorder. Had prior DUD treatment.)
- “I see the benefit of having somebody else who’s maybe more focused on either the treatment or the app itself...I don’t think the primary care [provider] needs to be concerned with that. They need to have some knowledge of it, but I think the major, the main focus of it would be with the social worker, plus the social worker would be able to follow up with the patient as far as once they get started using the app. It would be easier follow up for the social worker than the primary care.” (P5, participant with stimulant use disorder. Had prior DUD treatment.)

**Patients Felt a Tension Between Effectiveness and Convenience in Aspects of Workflow Design**

- “Frankly I don’t have a lot of time, so if I could do it over the phone I would...But I think this one [an in-person visit] would be more effective for a lot of people.” (P2, participant taking buprenorphine whose opioid use disorder is in remission. Had prior DUD treatment.)
- “Probably I have a slight preference for an in-person visit, just on the basis that I definitely have an easier time talking with someone in-person than over the phone. But yeah, in-person would work a little better in that sense, but on the phone is also very convenient.” (P10, participant with cannabis use disorder. No prior DUD treatment).

**The Workflow Needs to Meet Patients Where They Are At**

- “The first [phone call] I’d even say like within three days. Because if I go to the doctor and you gave me a screening and realize I’m an addict, you give me this thing, I actively want to make a difference, and the next day ...hang out with some friends and do coke, I’m not going to remember that. So two or three days later I would be totally–I wouldn’t be annoyed by that...And then like every week after that, just as a – hey, I’m here. I’m that app, remember? But the likelihood of me actually going home and doing it right away, probably very little. I would either have to get worse in whatever I was doing, or maybe the day after, on that terrible hangover, you’re like God, I need help – that’s when I’d probably look at it, or like look for it and try and find it.” (P4, participant with cannabis and stimulant use disorder. No prior DUD treatment.)
- “if it seems like I’m on track and using the app more often, then I wouldn’t need as many reminders, but if I’m off track, then it would be more helpful for someone is keeping up with me more constantly to hold me to it.” (P10, participant with cannabis use disorder. No prior DUD treatment.)

#### Established Relationships and Trust

Participants told us that they placed value in having a connection with the health care professional who worked with them on using a digital treatment. It would be ideal if this would be someone who had already established a relationship with them, regardless of their clinical role. Even if there was no pre-existing relationship, it was critical for this person to be compassionate and caring because conversations about substance use can be stigmatizing or embarrassing.

#### Openness to a Team-Based Approach

Although participants had a desire to work with someone with whom they had an existing relationship, the trust between a PCP and a patient appeared to extend to the broader primary care team. That is, a PCP would not necessarily be the one *holding [their] hand* throughout using an app. Some noted that PCPs are busy and often delegate care to other team members, including DUD care. However, there were some important bounds around this division of responsibility. For instance, having a “third party” from outside the health system support patients was seen as a bad idea.

An important exception to this theme is that a participant firmly wanted to work with one clinician. Needing to talk to an additional person about an app could be an “extra step” and could “add like the risk that they won’t do it at all.”

#### Tension Between Effectiveness and Convenience

Participants described the pros and cons of the intensity of hypothetical workflow interactions. Regarding the general workflow design, they noted that convenience and effectiveness were in opposition. In-person visits were more “personable” and would hold them more “accountable” to using an app; however, an in-person visit would require more time, travel, and potentially copays. Secure messages, or receiving reminders through the app, were considered convenient because participants could read and respond whenever they wanted; however, they acknowledged that these messages might not be as powerful as a phone call or face-to-face visit in keeping them engaged.

#### The Workflow Needs to Meet the Patient Where They Are At

Participants suggested that workflows should be tailored to individuals depending on their level of motivation, how often they use substances, and how long or how successfully they have been managing their substance use. For instance, this desire for tailoring reflected that someone with less motivation may need more hands-on help, someone who is using substances daily might want to be contacted every day, and someone who is just beginning to address their use and/or who is actively struggling may need more frequent follow-ups.

### Synthesis

Upon synthesis of the qualitative data, and considering the number of participants who preferred each storyboard scenario (Multimedia Appendix 1), we suggest a general approach for facilitating the use of DUD treatment apps, which is described using the story of a fictitious patient (Table 1). Briefly, once the patient expresses interest in using an app for DUD, the PCP confirms their willingness to talk with a clinician with mental health expertise that will help them get started and keep them accountable while initiating treatment. The clinician would teach the patient about the app, help them plan for using the app, agree on a structure for follow-up, and then provide regular follow-up by telephone and messaging (bold boxes, Figure 1). The clinician would further build rapport and ask the patient their preferred cadence, method of follow-up, and modality of contact (eg, in person vs phone). Importantly, the specific approach would remain flexible and tailored to the patient.

**Table 1:** A general approach for supporting patients in using a drug use disorder treatment app in primary care, based on participant preferences.

Workflow stage	Hypothetical experience for a fictitious patient *Cory*	Why participants liked this experience	Other experiences preferred by participants
Introduction stage: learning about the treatment app	Cory completes an annual health screen that asks about alcohol and drug use. Cory’s PCP^a^ expresses concern that her regular substance use could affect her health. Cory is interested in learning about options that could help her change. She agrees to talk to a mental health clinician on the primary care team. Privately, Cory and the mental health clinician discuss Cory’s goals for change, and review a few different options, including a treatment app for drug use.	Participants wanted to discuss substance use with a provider they already knew—but they also recognized that their PCP might not have the time or right expertise. Being seamlessly connected to a mental health clinician would feel “*more personal”* and provide support beyond what their PCP could offer.	Participants who wanted to talk to only one person—usually their PCP—said they would try to set up the treatment app on their own after their PCP ordered it.
Setup stage: getting started with the app	A mental health clinician describes features and content of the DUD^b^ treatment app that might be helpful to Cory. They give Cory instructions for how to get started, and they agree to check in after a couple weeks.	Most participants wanted to learn from someone on their care team how an app would benefit them and how to use it. Chances of using the app would be higher if these were discussed when motivation was high.	Several participants felt comfortable getting started with a treatment app on their own. Some said that technical support would be necessary.
Follow-up stage: getting support over time for engagement and executing a care plan while using the app	Cory gives the app a try. She eventually stops using the app after a couple weeks. However, she re-engages with the app after exchanging secure messages with her mental health clinician that covers a status update, tailored recommendations for using the app, and plans for a follow-up phone call.	Participants said that phone follow-up offered more support than secure messages and placed fewer demands on their time or finances than an in-person visit. Many wanted follow-up spaced out over time to help hold them accountable to using the app.	Benefits of follow-up via secure message include the choice in when and how often to responded to messages (unlike visits or phone calls). In-person appointments would be reserved for additional support and accountability.

^a^PCP: primary care provider.

^b^DUD: drug use disorder.

## Discussion

### Principal Findings

This study conveyed patient perspectives on the use of apps as part of treatment for DUD in primary care. Overall, participants desired to receive support from their health care teams in using apps and voiced little interest in using them without clinician guidance. There was a consensus among participants that they preferred to work with a trusted, competent clinician who could guide them in using the app over time. They stressed the importance of follow-up and felt that in most cases, this could generally be done through telephone with the addition of asynchronous secure messaging whenever needed.

These findings contribute novel information about patient preferences, laying the groundwork for research on the implementation of apps for DUDs in health care. Patients desire low-barrier, nonstigmatizing interventions for DUD in primary care [69,70]. Apps could potentially help address this gap in care. Although prior clinical trials have relied on research staff to “*train*” patients in using apps or facilitate their ongoing use [15,21,71-74], future studies can use these findings to inform the involvement of health care teams, instead of researchers, in various aspects of app delivery and implementation.

Overall, participants expressed that the most ideal way to offer apps for DUDs was during routine clinical interactions regarding drug use. This approach enables several aspects of care that participants desired, including the ability to build trust with a clinician, obtain medical and mental health advice, and go home with an appropriate app and clear expectations about treatment. The literature has described an alternative approach, where apps are offered as a self-guided option in the absence of clinician guidance [14,75], potentially reducing health care costs and burden. Such an approach was generally not preferred by participants in this study; some wanted access to a self-care option, but clinician-guided care was viewed as more effective and engaging. The findings are consistent with the literature that describes the need for *supportive accountability* while delivering app-based treatments, where patients establish a relationship with a helper that, among other things, sets expectations for app use and provides assistance over time [76]. We note that this study excluded participants whose drug use did not lead to a DUD; perhaps, self-care options should be further studied among patients with lower drug use severity.

Participants felt that treatment with an app should be seamless, free of technical glitches, and other barriers that could decrease their motivation or lead to “*getting stuck*.” Apps and associated clinical workflows need to impose few barriers. Indeed, many people with DUD report logistical barriers to treatment [77-82]. Some participants were also concerned about copays, which contributed to their support preferences. Future studies should design app implementation approaches that address socioeconomic and other barriers that could lead to the inequitable provision of these treatments [83,84].

The literature has also highlighted potential logistical challenges from a health system perspective. For instance, PCPs are heavily burdened and busy [27-29]. Indeed, a prior implementation study of a DUD app had to modify its initial plans by not involving PCPs [51]. Fortunately, participants in this study acknowledged these difficulties and were open to working with team members other than their PCP. Some studies in primary care failed to adequately engage patients in app use over time [21,22], and/or have been halted because workflows were not adequately developed to reach and communicate with patients [23]. This study advances the literature by adding patient voices that can inform future research on app delivery and implementation in routine care.

### Limitations

This study has several limitations. The sample size was small, which limits generalizability; however, we followed the standard of saturation in qualitative research. Furthermore, our use of purposeful sampling promoted a rich accounting of a diversity of patient perspectives and preferences across important demographic and substance use characteristics to inform the implementation of apps. Nonetheless, the small sample size limited our ability to identify the ideas and preferences of all possible patients. Although there was saturation in the analysis of workflow design principles and cross-cutting themes (eg, new data analyzed were repetitious of prior data and there was no indication of new emerging themes), the ideas regarding *tools* remained diverse. In this case, we opted to present a table that captured all participants’ ideas. Future study iterations are driven by additional data collection. Participants were from 5 clinics of a single integrated health care system in the United States from a single geographic region. Participants may have been more accustomed to working with multiple care providers than patients who received care in health systems with little care coordination. Thus, the results might not translate to patients from other groups. Although the goal of this study was to understand patient perspectives, clinicians’ voices were not assessed, which is also a limitation. This study’s focus on patients was driven by knowledge that variations in patient engagement impacted the results of trials of digital treatments [21,22,24-26,73]. Future research should elicit clinician perspectives on the mechanics of delivering apps for DUDs in primary care.

### Conclusions

The perspectives of primary care patients with cannabis, opioid, and stimulant use disorders suggest that a user-centered implementation of DUD treatment apps in primary care will require health systems to guide and support patients. Research is needed to evaluate clinician perspectives on workflows for delivering apps and to test the feasibility of the design considerations suggested by participants in this study. One such study is our ongoing implementation research trial funded by the National Institute on Drug Abuse (award number R01DA047954), which uses a randomized factorial design to evaluate different approaches for implementing a digital treatment for substance use disorders, while concurrently collecting qualitative data on implementation and workflow design. As more emphasis is placed on the use of digital treatments for DUDs for primary care patients, convenient methods for engaging patients and supporting them before, during, and after treatment will be paramount.

## Appendix

Multimedia Appendix 1 Interview materials and additional results for the study. Approaches for implementing app-based digital treatments for drug use disorders into primary care: A qualitative, user-centered design study.

## Abbreviations

DUD: drug use disorder
PCP: primary care provider
PI: principal investigator
