# Supplementary material for: Approaches for Implementing App-Based Digital Treatments for Drug Use Disorders Into Primary Care: A Qualitative, User-Centered Design Study of Patient Perspectives
Source: J Med Internet Res. 2021 Jul 6;23(7):e25866. doi: 10.2196/25866 (PMC8293157; doi:10.2196/25866)
Supplement: Multimedia Appendix 1 [file jmir_v23i7e25866_app1.docx]

# Interview materials and additional results for the article “Approaches for implementing app-based digital treatments for drug use disorders into primary care: A qualitative, user-centered design study

## Contents

[**Supplement 1.** Participant recruitment flow diagram 2](#_Toc65762427)

[**Supplement 2.** Participant Characteristics 3](#_Toc65762428)

[**Supplement 3.** Personas and Storyboards 4](#_Toc65762429)

[**Supplement 4.** Interview Guide Summary 11](#_Toc65762430)

[**Supplement 5.** Perspectives regarding the “people” who could facilitate a drug use disorder (DUD) treatment app in primary care. 14](#_Toc65762431)

[**Supplement 6.** Additional representative quotes illustrating cross-cutting themes 16](#_Toc65762432)

**Supplement 7.** Storyboard preferences  [20](#_Toc65762433)

# **Supplement 1.** Participant recruitment flow diagram


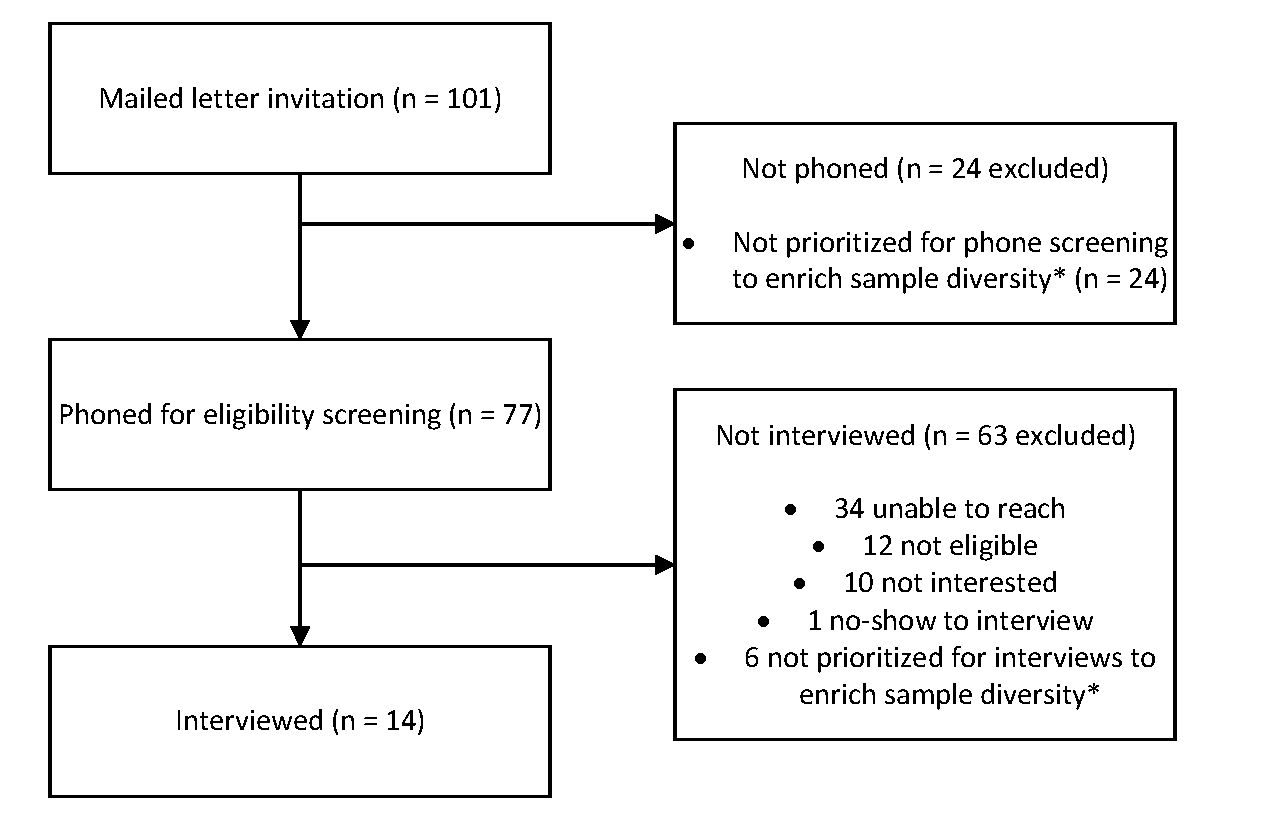


*Using the method of purposeful sampling, 24 patients were not phoned, and 6 patients were not scheduled for an interview to enrich sample diversity by race, gender, age, substance type (opioid, cannabis, stimulants), and prior treatment status.

# **Supplement 2.** Participant Characteristics

| **Supplement 2 Table.** Participant Characteristics | |
| --- | --- |
| Characteristic | *n* *or* M (range) |
| TOTAL | 14 |
| Gender |  |
| Female | 2 |
| Male | 10 |
| Transgender, gender non-confirming | 1 |
| Declined to state | 1 |
| Age in years; M (range) | 40.1 (19-65) |
| Person of color | 5 |
| Education beyond high school | 4 |
| Currently employed | 9 |
| Opioid use disorder | 6 |
| Cannabis use disorder | 9 |
| Stimulant use disorder | 4 |
| Prior specialty addiction services (any) | 9 |
| 12-step attendance | 4 |
| Professional/formal treatment | 7 |
|  | |

# **Supplement 3.** Personas and Storyboards

**Personas**

Patients were presented one of three personas describing a hypothetical patient that might be interested in a digital treatment app for drug use disorder:

1. Persona depicting a patient with cannabis use disorder symptoms

**Cory**

Cory is a primary care patient. She regularly uses cannabis. She continues using cannabis even though she has noticed it is causing health problems, including difficulty with remembering things. She has gradually used more and more to feel its effects. The few times she tried to stop, she was unable to make a change. Using cannabis is taking up a lot of time, which is affect­ing Cory’s relationships with family and close friends and her ability to perform at work. She is not looking to make a change right away, but she is open to learning about options for help.

1. Persona depicting a patient with opioid use disorder symptoms

**Olivia**

Olivia is a primary care patient. She regularly uses opioids. She continues using opioids even though she has noticed it is causing health problems. She has gradually used more and more to feel its effects. Using opioids is taking up a lot of time, which is affecting her relationships with family and close friends and her ability to perform at work. Olivia is looking to make a change right away, and she wants to get on a medication that will help.

1. Persona depicting a patient with stimulant use disorder symptoms

**Sid**

She regularly uses stimulants. She continues using stimulants even though she has noticed it’s causing health problems. She has gradually used more and more to feel its effects. The few times she tried to stop, she was unable to make a change. Using stimulants is taking up a lot of time, which is affecting Sid’s relationships with family and close friends and her ability to perform at work. Sid is not looking to make a change right away, but she is open to learning about options for help

**Storyboards**

*Storyboard Design Process and Rationale.* The study team held a series of participatory workshop meetings to design the storyboards. The goal was to depict core features of candidate approaches for offering and supporting the delivery of app-based treatments in primary care. Workshop members were from multiple disciplines (medicine, social work, informatics, design, public health), included clinician-researchers, and all had previously conducted service design or implementation research in primary care (e.g., integration of alcohol and drug services, treatment apps). This team elicited feedback on storyboard drafts from clinical leaders who had experience implementing a digital treatment for depression in primary care, and from a buprenorphine prescriber at a safety-net health clinic.

The storyboards depicted three workflow phases: 1) introducing the app to patients and helping them learn about it (“Introduction”); 2) for patients who are interested in the app, setting them up with the treatment (“Setup”); and 3) following up with patients who agree to use the app to promote engagement and to execute a care plan that includes the app (“Follow-up”).

Each workflow phase had 3-4 alternate scenarios. Scenarios varied in whether interactions were done virtually or in-person, how much help the patient received, which primary care team members were involved, and other aspects of communication and workflow.

The introduction phase scenarios began with the identification of the patient’s substance use with a screening instrument, as is recommended in primary care [1, 2]. The storyboard contained four alternative scenarios for the introduction phase:

- The primary care provider uses a “warm hand-off” to introduce the patient to an integrated mental health specialist, who provides information about the app. A warm hand-off is a common technique where certain care responsibilities are transferred to another primary care team member or specialist in front of the patient [3].
- An integrated mental health specialist proactively phones patients to introduce the app. Proactive population-based outreach is common in care management programs for chronic disease [4].
- The primary care provider provides information about the app directly to the patient.
- A patient learns about the app via a pamphlet. Patient health information materials like pamphlets are ubiquitous in primary care practices [5].

The setup phase scenarios were designed to obtain feedback about the extent to which patients wanted help with initiating the app after they expressed interest in using it. The scenarios varied in the amount of interaction with their health care team and the format (e.g., in-person versus phone). There were three setup scenarios:

- During an in-person visit, a primary care team member describes features of the app and provides a handout with download instructions . The patient is provided with a plan for follow-up.

Over the phone, a primary care team member makes a plan with the patient and sends instructions over secure message.

The patient is provided instructions for downloading the app to set it up on their own.

The follow-up phase scenarios were designed to elicit preferences regarding the format of follow-up. There were three follow-up scenarios, all of which included some communication over secure message:

- Follow-up in-person at the clinic
- Follow-up over the phone
- Follow-up by secure message only

*Storyboard administration.* Interviewers read the storyboards to participants. After presenting all scenarios within a phase, interviewers asked patients to pick one or two scenarios that they preferred the most, rank them by preference, and then probed for additional information including reasons for preferred and non-preferred scenarios. Below is an example storyboard for a hypothetical patient, Cory, who has cannabis use disorder. We note that while the print interview materials used the title “social worker”, participants often did not know the duties of a primary care-based social worker, so interviewers provided clarification and used other titles as needed.

**Introduction Scenario 1: Primary care provider introduces social worker**


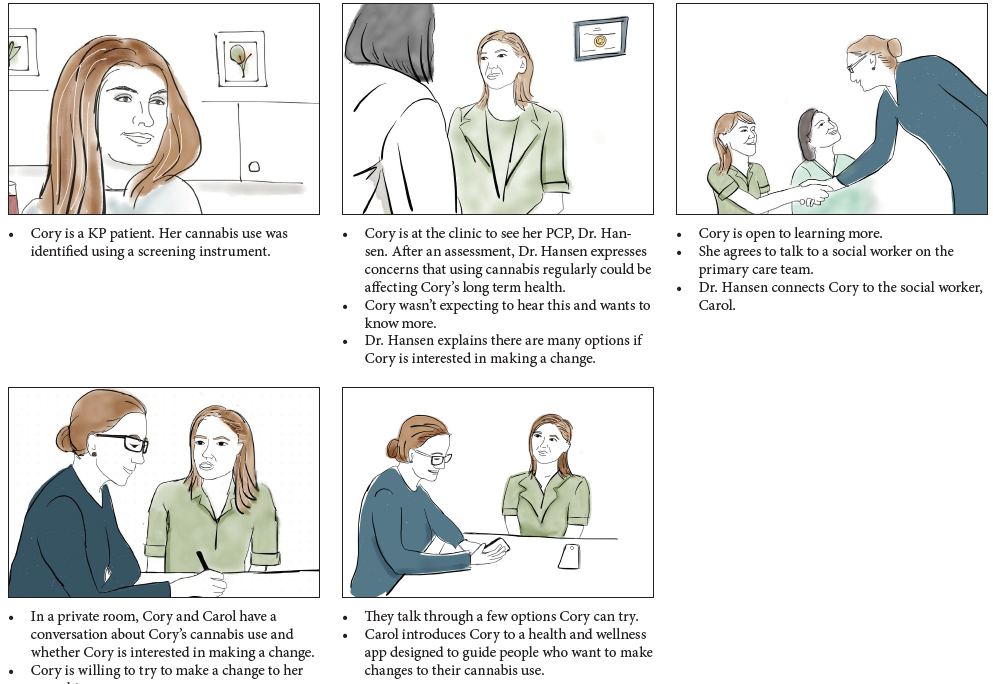


**Introduction Scenario 2: Phone outreach**


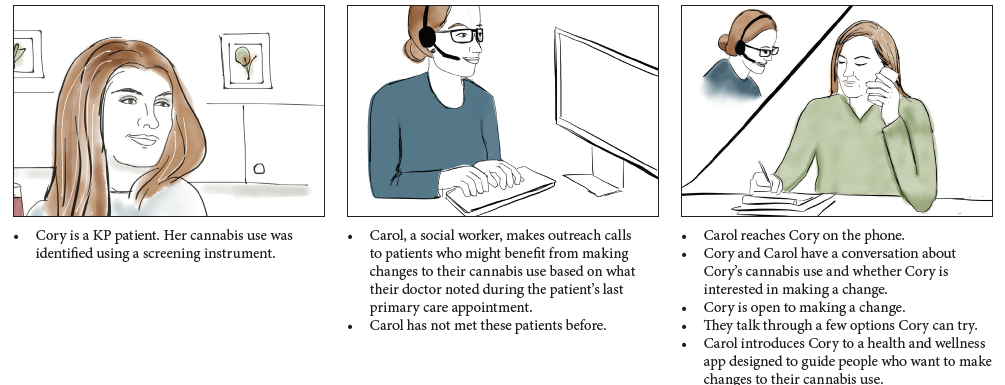


**Introduction Scenario 3: Primary care provider places order**


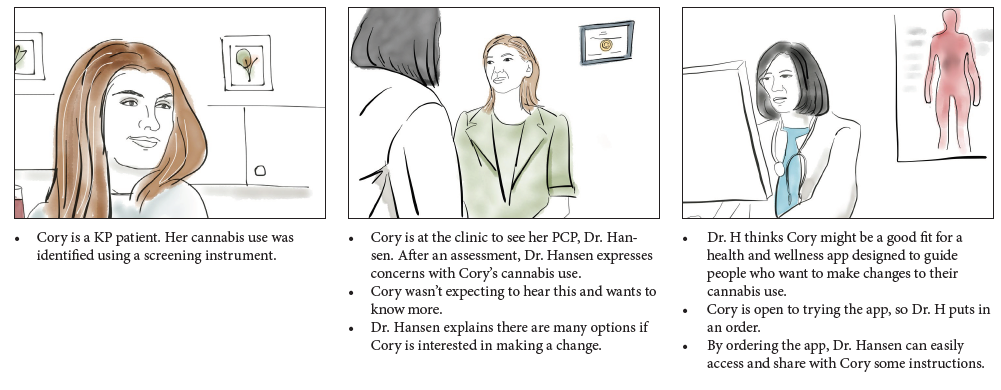

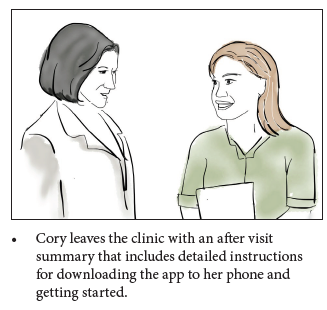


**Introduction Scenario 4: Self-referral**


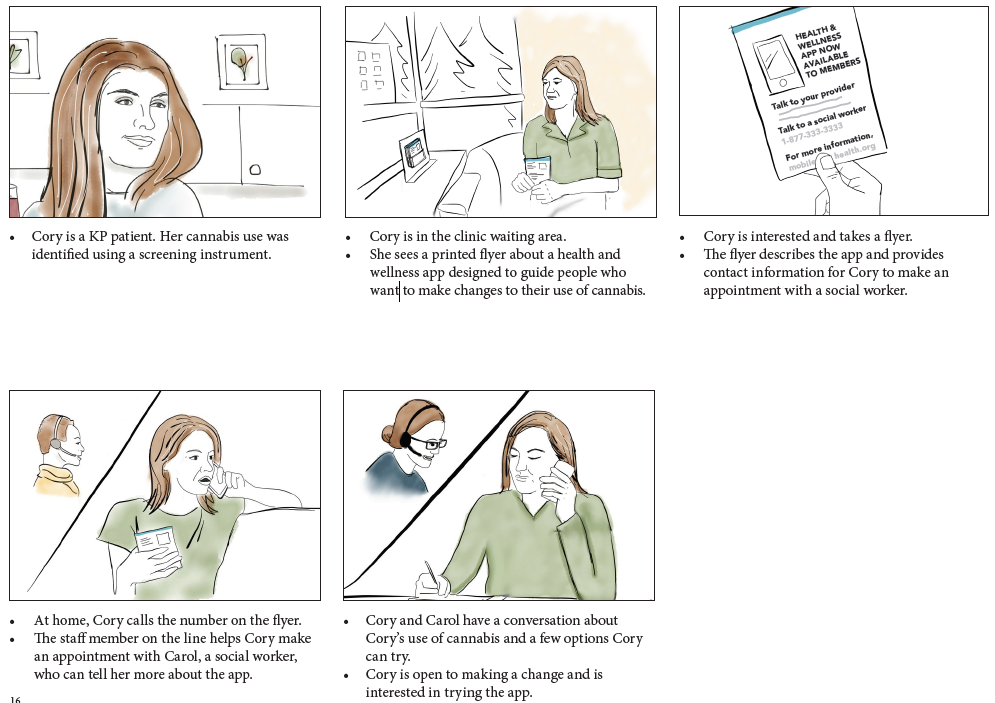


**Setup Scenario 1: In-person setup and plan**


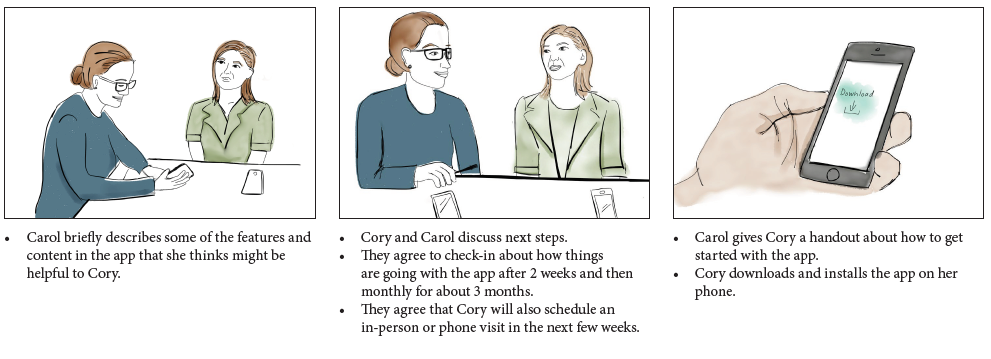


**Setup Scenario 2: Phone call setup and plan**


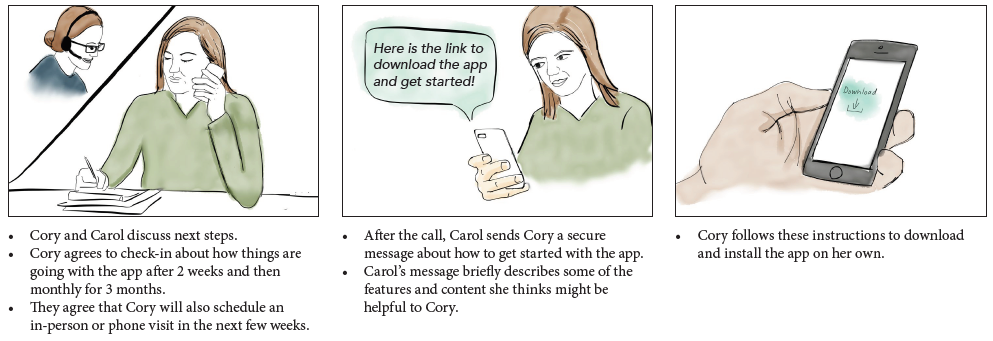


**Setup Scenario 3: Solo setup**


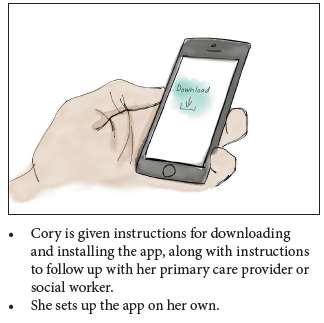


**Follow-up Scenario 1: In-person and secure messages**


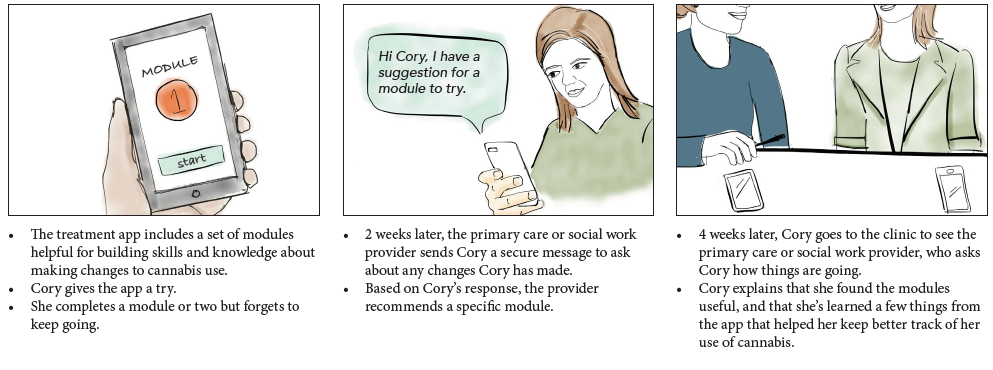


**Follow-up Scenario 2: Phone and secure messages**


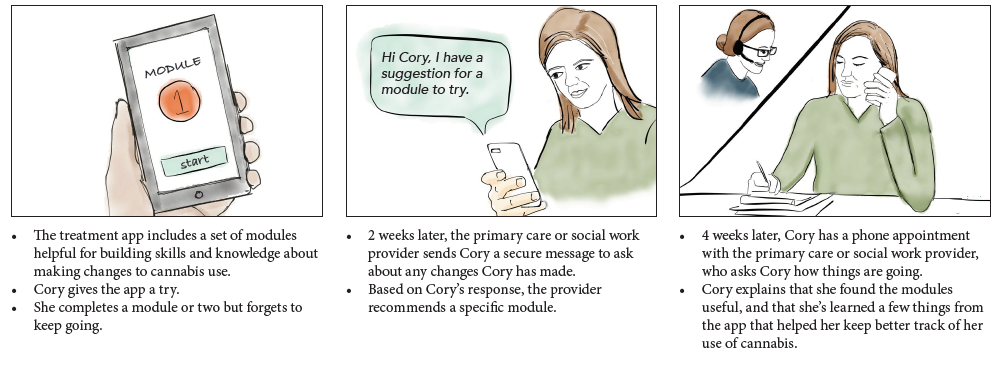


**Follow-up Scenario 3: Secure messages only**


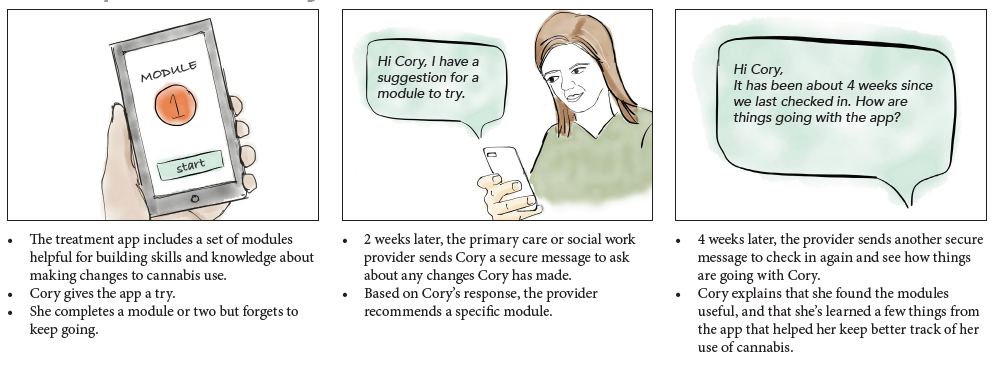


# **Supplement 4.** Interview Guide Summary

**Introduction to the Study**

Several new health and wellness apps incorporate tools that research has shown to be helpful for people who might want to change their substance use. By substance use we mean taking drugs or using medicines without a prescription, or in ways that are not prescribed. These health and wellness apps, which can be accessed by a computer or smartphone, help people explore their values, keep track of their drug use or drinking, set measurable goals, learn skills for problem solving and dealing with negative thoughts, and replace unhealthy habits with healthier ones. Some of these apps reward people for successfully learning strategies for reducing their substance use. We are interested in learning the best ways to offer these types of apps to people who might want to make changes to their substance use.

**Background Information: Patient experiences in using take-home/use-at-home patient materials**

1. We are interested in how patients react to being asked to “take stuff home” with them to use, like a blood pressure monitor, a glucose monitor, a Fitbit monitor, or an “app”… or perhaps even informational pamphlets abut diet or exercise. First off, have you ever been given something like this to use at home?  If yes, how’d it go?
2. In your mind, what was important about what they said?

| Probes:   - - What was it (i.e., the material/tool)?   - I’m interested in learning about what health care providers say to patients when they ask them to use medical devices or other materials at home. Some examples are blood pressure monitor, a CPAP, a glucose monitor, educational pamphlets, or any wearable devices (Fitbit, etc.). Tell me about anything, other than medications, a health care provider has recommended for you to use at home for any health concern. - In other words, I’m interested in how the health care provider discussed the ___ with you, I’m not interested in the ___ itself. What did the health care provider say to you when they recommended that you use the __? - Tell me about what they said that you thought was important. what worked well? What didn’t work well? - If you have not been recommended a take-home tool/material from a healthcare provider, what kind of tools have you used or would you consider using? Would you consider using an app/online tool (why or why not?)? |
| --- |

1. Tell me about any websites or apps a health care provider has recommended to you.

- Can you tell me little bit about how well they worked?
- What worked well, what didn’t work well, can you give me any examples

1. Thinking back to your previous experiences, what has prevented you, or what would prevent you from using an app or something else recommended by your healthcare provider?
   - In circumstances like this, what could your health care provider do to help you keep using it?

**Background Information: Current SUD care experiences, attitudes, expectations, needs**

1. To what extent have you talked about your substance use with your health care provider, either them raising it with you or you raising it with them?

- What recommendations have you received to make changes in your substance use?
- What was your initial reaction to what they said? How has that changed, if at all?
- What was helpful about the conversation?
- What else would you have liked or needed in that conversation?

**Workflow: Patient preferences for potential workflows for using apps**

*Present Persona and Storyboard*

- Next, we want to understand your preferences for clinical support or guidance when learning about and using an app designed to guide people who want to make changes to their substance use.
- We are going to go through a series of storyboards. If you are not familiar with storyboards, they are like comic strips that are meant to help people visualize a new health care experience so that we can get feedback on specific aspects.
- We’ll go through the storyboards like a “choose your own adventure” activity where we will focus on parts of the experience.
- I will show you several options and ask you to indicate your preferences.

**Introduce persona**

- [Present persona] Before we look at the storyboards, we want to introduce our character, _______. [read the character description].
- As we go through the storyboards, try to put yourself in ____’s shoes and respond from your own perspective as you describe your preferences.

[As the interviewer walks the participant through each dimension of the workflow, the participant is asked to indicate their preferences for scenario variations. For example, if the participant selects “Intro A: Warm hand-off” as a preferred start to the workflow, they will then indicate which of the scenario variations they prefer for the next step in the workflow, and so on. Throughout this process, the interviewer will ask questions (from “Storyboard questions” below) about preferences for each dimension. For instance, when discussing the 4 possible scenarios related to The Context of Introduction, the interviewer might ask, “How would you want this app introduced to you?”.]

**How to describe health and wellness apps when presenting the storyboard:** “These types of apps use tools that research has shown to be helpful for reducing substance use. Some help patients explore their values, keep track of their drug use and drinking, set goals, learn skills for problem solving and dealing with negative thoughts, and replace unhealthy habits with healthier ones.”

General guidance:

- Have participant choose two that they would prefer. Why are these preferable?
- Then narrow down to their most preferred option.
- Once they have gone through the three scenarios, walk through the storyboard that they chose.

*Storyboard questions*

**Introduction scenarios:** The first set of storyboards that we are going to focus on is the introduction—how you might learn about an app designed to guide people who want to make changes to their substance use.

Read through each option.

1. How would you want to learn about an app designed to help you make changes in your substance use?

- How would you want this app introduced to you?
- Who on your primary care team would you want to introduce it to you?
- Are there other ways you see this working?

**Setup scenarios:**  For the second set of storyboards, we are interested in knowing how much support you would want/need when getting started with the app.

Read through each option.

1. What would you need to know when starting to use an app like this? [technical guidance]

- What kind of orientation would you want to have? [orientation on what the app offers]

1. What would be the best way to get help using the app if you got stuck?

**Follow-up scenarios:** For this last set, we are going to ask for your preferences around follow-up when using the app.

Read through each option.

1. What kind of follow-up would you want by your primary care team to help you keep using an app?

- How often should they contact you?
- How should they contact you?
- In what format (over the phone, in-person, a secure message)
- How might you involve their family members?
- Who on the primary care team would you want to follow up with? [potential probes: peer, nurse care manager, health coach; how would this role differ at different stages of treatment or recovery]
  1. What are your thoughts on a different person on the care team following up with you after the initial introduction to the app?
- How much would cost/a copay be considered in this follow up?

**Questions once storyboard scenarios are picked:**

1. How well overall do you think the experience reflected here would work for you?
2. What changes to the storyboard would you suggest to make it work for you?
   - If you were to think about this scenario for [any other substances that apply to participant], what changes, if any would you make to this scenario?
3. Would you see yourself using an app that research has shown to be helpful for reducing your substance use?

| Probes:   - Why/why not? - In an ideal world, what would the app look like? What would be useful for you? [Prompt: exploring your values, keeping track of your drug or alcohol use, setting measurable goals, learning skills for problem solving and dealing with negative thoughts, replacing unhealthy habits with other ones, and rewarding people for learning new strategies.] - How many days per week would you use it? - How many hours per day? - If you were to think about an app for [any other substances that apply to participant], what would be different about the ideal app? |
| --- |

1. Some apps are designed to supplement treatment that is led or supervised by a clinician. Other apps are designed for patients to use on their own, without the support of a clinician. Tell me about your thoughts about which would be the best fit for you.
   - What kind of clinician would you prefer?

# **Supplement 5.** Perspectives regarding the “people” who could facilitate a drug use disorder (DUD) treatment app in primary care.

| **Supplement 5 Table.** Preferences regarding “people”—including clinicians and non-clinicians—who could support the introduction, setup, and follow-up a drug use disorder (DUD) treatment app in primary care | | |
| --- | --- | --- |
| Type of clinician or non-clinician | Reasons why patients preferred to work with these individuals | Other considerations stated by patients |
| Social workers or other clinicians on the primary care team who are designated mental health experts | - May have more experience treating addiction, more knowledge about treatment options or tools [Introduction, Follow-up] - May be able to spend more time describing features of a treatment app and guide patients through the set-up process [Introduction, Setup] | - Preconceived ideas about the role of these clinicians (e.g., they are only involved in “serious issues,” or they will remove children from families) [Introduction, Follow-up] - Stigma surrounding the term “social worker” [Introduction, Follow-up] - Lack of experience working with these clinicians [Introduction, Follow-up] |
| Primary care providers (PCPs) | - Trusted to give medical advice [Introduction, Follow-up] - An established relationship with the P­­CP could make patients feel more comfortable talking about drug use [Introduction] - Working solely with the PCP would have benefits: involving too many other people makes care feel “cold” and “detached” [Introduction, Follow-up] | - Best at overall health care but could know less about treating addiction [Introduction, Follow-up] - Logistical constraints; they may not have time or knowledge to walk patients through app setup [Setup] - Follow up appointments may be harder to schedule (F) |
| Other clinicians in primary care (e.g., nurses on the primary care team) or elsewhere (e.g., therapists, addiction specialists) | - May have established relationships with other clinicians [Introduction, Follow-up] - May have previously discussed drug use with other clinicians (e.g., a counselor) [Introduction, Follow-up] - Would be convenient to work with another clinician who they already know [Introduction, Follow-up] | - None stated* |
| Other people with DUD | - Interested in learning about apps from other patients who have or previously had DUD; they have “been through it.” [Introduction] - Their recommendation would be helpful in deciding whether to use an app [Introduction] | - More useful for initially learning about the app, peer involvement was seen as less important for helping with ongoing app use [Introduction, Setup, Follow-up] |
| Information technology (IT) specialists and staff at software companies | - Felt comfortable calling or messaging an IT specialist or developer for answers to technical questions about downloading, setting up and using a DUD treatment app [Setup] - Clinicians may not know how to resolve technical questions; technical questions would use up valuable visit time [Setup] | - Wanted assurance that treatment app companies would be bound by HIPPA [Introduction, Setup, Follow-up] - Patients differentiated between logistical/setup questions, which they would be comfortable asking an IT specialist, and treatment-related questions, which they would want to talk to their clinician about [Introduction, Setup, Follow-up] |

Some statements relate to only one workflow stage, others are more generic to the entire process. Words in square brackets note the most applicable workflow stage. *We did not consistently ask patients about professionals in this row; however, some patients volunteered reasons why patients might want them involved.

# **Supplement 6.** Additional representative quotes illustrating cross-cutting themes

| **Supplement 6 Table.** Themes derived from analyses across the codebook: Cross-cutting recommendations for designing a patient-centered approach for offering treatment apps for drug use disorders (DUD) in primary care | |
| --- | --- |
| **Theme and Representative Quotes** | |
| **Established relationships and trust** would facilitate a better patient experience | |
|  | It's a person who's already talking to her about her drug addiction, like supplying Suboxone or whatever, that is saying hey, this might help. And I feel like people would be a little bit more receptive if it's someone that they already trust with their treatment. –**P1, Patient with cannabis and opioid use disorder. Had prior DUD treatment.** |
|  | I mean the main thing is support… I would say the best people that will help you is if they understand what you're going through, especially during that period, and they need to understand what you went through. It doesn't necessarily have to be that way, but that's how I was, and primary care provider helped me in the professional way, and then the emotional issues of that time … But I will say if someone is truly trying to help this person making them know that there's still someone that cares about them, whatever, in any way it's a good thing. It is. Some people have no one, and even small interactions can make a difference in positive ways. –**P2, Patient taking buprenorphine whose opioid use disorder is in remission. Had prior DUD treatment.** |
|  | I know for me personally that if I'm talking with somebody – I mean, if you can send me something, I can more likely put it on the back burner. Whereas if I have an actual conversation with them – I don't know, it's just more personal. I think it's more of a personal check in, that there's a real human being right there that's interested in my care –**P3,** **Patient taking buprenorphine whose opioid use disorder is in remission. Had prior DUD treatment.** |
|  | As we go through this, I'm realizing a great deal of my apprehension about some of these things is the embarrassment about being addicted to something. Which is that's just social stigma and I'm very open about my drug use with my friends, and it's not like a – I don't think anyone would think less of me for doing it, but there is a weird, like low key embarrassment, that I have to ask someone else for help. That's why I hesitated for the first one, with having her like sit down with me. It would be embarrassing, but as long as that person was compassionate and like actually cared, the embarrassment would go away, because I actually need help. **– P4, Patient with cannabis and stimulant use disorder. No prior DUD treatment.** |
|  | Not having met them before, to me [a phone outreach] would be more difficult – to me a phone outreach would be better if I've already met and talked with the individual before. Just getting a call out of the blue from somebody, I would be a little reticent as to providing either a lot of information or being totally honest and accurate with things … After the introduction, yes, it would be very helpful, but just to begin with by phone? No. – **P5, Patient with stimulant use disorder. No prior DUD treatment.** |
|  | I think it's just a little more personal and in that way you get to meet the social worker so you know who you're talking to, you can put a face with a voice. It's a little bit nicer that way, a little more personal. – **P6, Patient taking buprenorphine whose opioid use disorder is in remission. Had prior DUD treatment.** |
| Patients are **open to team-based approach** and not just help from a primary care provider | |
|  | I think it would kind of be more like a team effort, maybe the doctor kind of asks are you interested in this, yes/no. If I say yes, he's like oh, this person has more information and then that other person would be like Carol walking me through it. – **P7, Patient with cannabis use disorder. Had prior DUD treatment.** |
|  | I mean if there were actually social workers doing this, then I suppose that would be who I would be contacting, and I wouldn't really have any issue with that because that's just sort of how it would be. It would be another person I'm meeting to satisfy a different medical need that I have. I don't have a lot of hang-ups about meeting different providers for different issues. I've done a lot of that in the past. – **P8, Patient with cannabis and stimulant use disorder. Had prior DUD treatment.** |
|  | I mean, not a stranger. They're part of the care team. – **P9, Patient with cannabis and opioid use disorder. Had prior DUD treatment.** |
|  | Originally, I think you need to see your primary care person one-on-one to get the ball rolling, but then after that, if the introduction takes place at some point – introduce the social worker, exactly. And then she can call up and say hey, how's this app working? Would you try this module? Bla bla bla. Four weeks later get back with me and find out how things are going. I think it makes a lot of sense. I like it. I think it's kind of new wave thinking. **–P3, Patient taking buprenorphine whose opioid use disorder is in remission. Had prior DUD treatment.** |
|  | I see the benefit of having somebody else who's maybe more focused on either the treatment or the app itself … I don't think the primary care needs to be concerned with that. They need to have some knowledge of it, but I think the major, the main focus of it would be with the social worker, plus the social worker would be able to follow up with the patient as far as once they get started using the app. It would be easier follow up for the social worker than the primary care. – **P5, Patient with stimulant use disorder. Had prior DUD treatment.** |
| Patients felt a **tension between engagement and convenience** when they felt the easy option might not be enough to sustain engagement and/or lead to change in substance use | |
|  | Frankly I don’t have a lot of time, so if I could do it over the phone I would … But I think this one [an in-person visit] would be more effective for a lot of people. – **P2, Patient taking buprenorphine whose opioid use disorder is in remission. Had prior DUD treatment.** |
|  | It's a little harder for me to be as motivated to take it to the next step if I'm just bringing like a flyer home, I feel like I would go on that stack of papers with like the pamphlet and maybe never get revisited. I wouldn't necessarily be opposed to [PCP warm handoff to SW], but I guess sometimes I feel when I'm at the doctor and I've already dedicated a good chunk of my day to that whole process, because when I go in, I also have to go to the lab, leave a urine sample, so the whole process can eat up like a good two hour chunk of my time. So I probably wouldn't be as enthused about extending my visit or being introduced to another person during that visit, you know? I guess that's why the option of them reaching out over the phone would be something I'd do more, in line with, not opposed to. –**P9, Patient with cannabis and opioid use disorder. Had prior DUD treatment.** |
|  | Probably I have a slight preference for an in-person visit, just on the basis that I definitely have an easier time talking with someone in-person than over the phone. But yeah, in-person would work a little better in that sense, but on the phone is also very convenient. –**P10,** **Patient with cannabis use disorder. No prior DUD treatment.** |
|  | As long as I've actually talked to a real person. I would not do [phone follow up] if it had been the phone the entire time. Yeah, I feel like having made the contact and then that person calls me, I'd be more into that – **P4, Patient with cannabis and stimulant use disorder. No prior DUD treatment.** |
|  | I know sometimes I do better when I have other people around me telling me what to do, kind of. Not bossing around, but kind of pushing me? I think that works for most people. So like checking in with the social worker or health care provider might not actually be a bad idea.  **– P11, Patient with cannabis use disorder. No prior DUD treatment.** |
|  | [If] you're just doing secure messaging, it takes much less time and effort so you can do a lot more for the same amount of investment. I think without the personal aspect, keeping pressure on, the more frequent messaging would be helpful to keep a person using the app occasionally. In terms of completing a module and forgetting to keep going, notifications would be useful there. **– P12, Patient with cannabis use disorder and stimulant use disorder. Had prior DUD treatment.** |
| **The workflow needs to meet patients where they are at** | |
|  | The first [phone call] I’d even say like within three days. Because if I go to the doctor and you gave me a screening and realize I’m an addict, you give me this thing, I actively want to make a difference, and the next day … hang out with some friends and do coke, I’m not going to remember that. So two or three days later I would be totally – I wouldn’t be annoyed by that… And then like every week after that, just as a – hey, I’m here. I’m that app, remember? But the likelihood of me actually going home and doing it right away, probably very little. I would either have to get worse in whatever I was doing, or maybe the day after, on that terrible hangover, you're like God, I need help – that's when I'd probably look at it, or like look for it and try and find it. But this, I feel like having someone go through, actually physically download the app with me, go through it with me and then I leave full of knowledge and hope? That'd be great **– P4, Patient with cannabis and stimulant use disorder. No prior DUD treatment.** |
|  | If I had a specific problem with something, then yes, it would be good to have a follow up. It would depend on what we were talking previously. If there was something that was concerning, a follow up to that problem, whatever would be preferable. But it's just an everyday thing, there's nothing going on, no surprises, not necessary. **– P2, Patient taking buprenorphine whose opioid use disorder is in remission. Had prior DUD treatment.** |
|  | (describing preference solo setup) if I'm already getting to this stage in the process, I feel like I don't need another nudge as far as like taking the steps to download the app and install it and start using it. **– P9, Patient with cannabis and opioid use disorder. Had prior DUD treatment.** |
|  | Maybe comparing it to like other methods of rehab. You know, people go to A.A. meetings if they're alcoholics, some people might not be comfortable with that, so maybe to say maybe if you're uncomfortable with these type of methods, we have some new things called this app, bla di bla, you can recover in this time. You know? **– P11, Patient with cannabis use disorder. No prior DUD treatment.** |
|  | if it seems like I'm on track and using the app more often, then I wouldn't need as many reminders, but if I'm off track, then it would be more helpful for someone is keeping up with me more constantly to hold me to it. –**P10,** **Patient with cannabis use disorder. No prior DUD treatment.** |
|  | It could be once a day. It could be every five minutes. It depends on what you're trying to do. I mean, I've been there before, and I know what it takes, but I also know – it's hard, because I understand that side of it **– P14, Patient with opioid use disorder and stimulant use disorder. Had prior DUD treatment.** |
|  | One thing I've found with Dr. [LAST NAME] is that if she hasn't gotten a response to a text message that she sends me, that she does call me to say hey, I sent you this message, I haven't heard from you, and just wanted to follow up. So then we would discuss things right then and there. And I think that would be a good idea as far as this is concerned as well, that yeah, you could send a secure message, but depending on what you do or don't get in response to that, then you may want to move up the in person or phone conversation between the patient and primary care or social worker. – **P5, Patient with stimulant use disorder. Had prior DUD treatment.** |
|  | (describing preference for frequency of follow-up) Might have to be daily…. Because people use substances daily. **– P11, Patient with cannabis use disorder. No prior DUD treatment.** |

# **Supplement 7.** Storyboard preferences

| **Supplement 7 Table.** Storyboard preferences (n=14) | | |
| --- | --- | --- |
| **Phase and Scenario** | **Most preferred scenario** | **Second most preferred scenario** |
| **Introduction Phase** |  |  |
| Scenario 1: Primary care provider introduces social worker | 9 | 2 |
| Scenario 2: Phone outreach | 0 | 3 |
| Scenario 3: Primary care provider places order | 4 | 6 |
| Scenario 4: Self-referral | 1 | 1 |
| **Setup Phase** |  |  |
| Scenario 1: In-person setup and plan | 10 | 1 |
| Scenario 2: Phone call setup and plan | 2 | 2 |
| Scenario 3: Solo setup | 2 | 1 |
| **Follow-up Phase** |  |  |
| Scenario 1: In-person and secure messages | 3 | 2 |
| Scenario 2: Phone and secure messages | 7 | 3 |
| Scenario 3: Secure messages only | 4 | 0 |

In semi-structured interviews, interviewers read aloud the storyboards shown in Supplement 3. For each phase, participants picked one or two scenarios that they preferred the most then ranked them by preference. This table shows the number of participants that picked each scenario as their most and second most preferred options.

**References**

1. U.S. Preventive Services Task Force. Draft Recommendation Statement: Illicit Drug Use, Including Nonmedical Use of Prescription Drugs: Screening. 2019.

2. U.S. Preventive Services Task Force. Unhealthy Alcohol Use in Adolescents and Adults: Screening and Behavioral Counseling Interventions. 2018.

3. Agency for Healthcare Research and Quality. Warm Handoff: Intervention. Rockville, MD2017 [2/5/2021]; Available from: https://www.ahrq.gov/patient-safety/reports/engage/interventions/warmhandoff.html.

4. Agency for Healthcare Research and Quality. Care Management: Implications for Medical Practice, Health Policy, and Health Services Research. 2018 [2/5/2021]; Available from: https://www.ahrq.gov/ncepcr/care/coordination/mgmt.html.

5. Moerenhout T, Borgermans L, Schol S, Vansintejan J, Van De Vijver E, Devroey D. Patient health information materials in waiting rooms of family physicians: do patients care? Patient Prefer Adherence. 2013;7:489-97. PMID: 23766635. doi: 10.2147/PPA.S45777.
